# Supplementary material for: Pelagic fish predation is stronger at temperate latitudes than near the equator
Source: Nat Commun. 2020 Mar 31;11:1527. doi: 10.1038/s41467-020-15335-4 (PMC7109113; doi:10.1038/s41467-020-15335-4)
Supplement: Supplementary file 1 — Supplementary Information [file 41467_2020_15335_MOESM1_ESM.pdf]

SUPPLEMENTARY INFORMATION

Pelagic fish predation is stronger at temperate latitudes than near the equator

Roesti et al.

CONTENT

Supplementary Note 1: Consideration of alternative estimates of predation

Supplementary Tables 1 – 4

Supplementary Figures 1 – 22

Supplementary References

Supplementary Note 1: Consideration of alternative estimates of predation

Our default measure of annual relative predation summed monthly catch and effort data per 5°x5° grid cell and year to calculate annual 'nominal CPUE' (see Methods for further details). We tested the robustness of our results to alternative ways of calculating relative predation. We found that these alternatives yielded highly similar results to the ones obtained from our default metric of relative predation.

First, we assessed the impact of changing how relative predation was calculated per year and grid cell, and per five-year time interval. To generate the input data for our default GAMMs, we first calculated annual nominal CPUE (relative predation) per grid cell. (This involved taking the yearly *sum* of all predators caught divided by the yearly *sum* of all hooks set per grid cell, and then taking the five-year *median* across all these annual values for every grid cell.) We tested the effect of instead calculating relative predation based on (A) five-year *means* of annual nominal CPUE, and (B) five-year medians based yearly *means* of monthly nominal CPUE (Supplementary Table 2). GAMMs ran on data generated through these alternative calculations yielded highly similar latitudinal patterns to the ones obtained from our default calculation of relative predation (Supplementary Figure 16).

In the second approach, we tested the robustness of our results to different weighing of individual data records. Instead of calculating five-year medians of annual relative predation values, we conducted analyses in which we gave equal weight to monthly longline records (i.e., the individual data records provided by the longline fisheries datasets; Supplementary Table 2). We binned these individual data records by grid cell within consecutive 11-year time periods (working with 11-year periods ensured that sufficient data points per grid cell were available for this analysis). For all grid cell x 11-year time period bins with at least 20 records, we fitted a linear model with log-effort as the predictor variable and log-catch as the response variable (27% of bins did not have sufficient data). We then took the antilogarithm of the intercept from every log-log model as an alternative estimate of relative predation, with larger values indicating greater predation risk (Supplementary Figure 18). Results based on this alternative relative predation metric (hereafter 'intercept-based' (IB) relative predation) were similar to the annual relative predation reported in the main text. As before there was a significant effect of latitude on IB relative predation, with higher values in temperate regions than in the tropics (Supplementary Figures 19 & 20). Similar to results based on

annual relative predation, this basic pattern was consistent across time, despite an overall decrease in IB relative predation through time (Supplementary Figure 21). Finally, analyses based on IB relative predation recovered the same negative relationship between fish species richness and relative predation (Supplementary Figure 22). These results suggest that our conclusions are robust to different weighting given to individual fisheries records.

In the third approach, rather than standardizing total annual catch by the total number of hooks set per grid cell (see above), we ran GAMMs with the annual 'sum of fish predators caught' (catch) as the dependent variable and with annual 'sum of hooks set' (effort) as a fixed effect in addition to 'time' and 'latitude'. The syntax of these GAMMs was otherwise identical to the syntax used to run the default GAMMs (see Methods for details). The results from these analyses support the idea of a relatively greater number of fish predators at temperate latitudes than around the equator (Supplementary Figure 17), thus providing further support in favor of stronger relative predation in temperate regions than at the equator.

Supplementary Tables

Supplementary Table 1. Dataset sources and information. Note that the Pacific Ocean is split into two datasets, one representing the East Pacific and one the West and Central (hereafter simply 'West') Pacific. Because these datasets are curated by different commissions and differ in some aspects (see below), we analyzed these datasets independently. See the *c_DataPrep_allOceans.r* *R*-code (Supplementary Software 1) for details.

Dataset	Source (date accessed)	Dataset specific filtering strategy; other remarks
**Atlantic**	International Commission for the Conservation of Atlantic Tunas (Task II Catch and Effort statistics) https://www.iccat.int/Data/t2ce_20171120.zip (Feb. 2018)	Due to too limited spatial and temporal coverage, we removed all data entries recorded for latitudes higher than 60° (99 out of 152,915 individual data points) and lower than -50° (31 data points) from the dataset. Following data filtering, the 44.6% of the data records at 1°x1° resolution were collapsed to 5°x5° resolution
**East Pacific**	Inter-American Tropical Tuna Commission (PublicLLSharkNum.csv and PublicLLTunaBillfishNum.csv) http://www.iattc.org/PublicDomainData/IATTC-Catch-by-species1.htm (Feb. 2018)	There were only seven data entries for catch locations at latitudes higher than 45°, and one entry at a latitude below -45°. These data entries were not considered for further analysis. Data were recorded at a 5°x5° grid cell resolution. Other remarks: The dataset for shark catches, and the dataset for billfish and tuna catches were concatenated by time and grid cell prior to analysis. Notably, the shark dataset starts only in the 1970's (1979)
**West Pacific**	Western and Central Pacific Fisheries Commission (LONGLINE_60.DBF; one data file per decade) https://www.wcpfc.int/wcpfc-public-domain-aggregated-catcheffort-data-download-page (Feb. 2018)	For this dataset to align with the one from the East Pacific without overlap, all data for the longitudes 212.5, 217.5, 222.5, and 227.5 were removed. Due to limited resolution, data for latitudes below -50° (7 data entries only) and higher than 45° (2 entries) were removed
**Indian Ocean**	Indian Ocean Tuna Commission (IOTC-2017-DATASETS-CELongline.csv; one data file per decade) http://www.iotc.org/documents/ce-reference (Nov. 2017)	Data entries from several fleets were excluded entirely because data from these fleets appeared systematically as outliers (12% from the raw data). These fleets included 'SYC', 'MOZ', 'MUS', 'MYS', 'ZAF', 'AUS'. For example, all entries of the 'MOZ', ''MUS', 'MYS', and 'ZAF' fleets recorded zero catches despite a considerable number of hooks set. We further excluded data entries recorded at a coarser than 5°x5° grid cell resolution (0.06% of the remaining data), data entries deemed to be of poor quality (quality code <2; 10% of the remaining data) and data entries obtained from fishing with 'Exploratory longlines' and with 'Longlines targeting sharks' (0.7% and 0.3% of the remaining data, respectively). The 0.2% of the remaining data recorded at 1°x1° resolution were collapsed to 5°x5° resolution. For this dataset to align with the one from the West Pacific without overlap, all data for longitudes below 140 were removed. Data recorded for latitudes lower than -50° (3 data entries only) were removed

Supplementary Table 2. Summary of final longline fisheries data analyzed. Numbers reflect final numbers after filtering and removing extreme relative predation values (see Methods as well as *c_DataPrep_allOceans.r* *R*-codes provided as Supplementary Software 1 for details).

**Ocean**	**Maximal latitudinal range**	**Number of 5°x5° Grid cells**	**Number of individual catch/effort records** (from all months and years)*	**Number of annual relative predation estimates**	**Total Effort** (Cumulative number of hooks set)	**Total Catch** (Cumulative number of fish predators caught)
**Indian Ocean**	50°S – 30°N	251	72,575	9,899	1.12×10^{10}	1.81×10^{8}
**Atlantic**	50°S – 60°N	321	151,142	11,725	7.13×10^{9}	1.27×10^{8}
**East Pacific**	45°S – 45°N	229	69,198	8,536	7.86×10^{9}	1.25×10^{8}
**West Pacific**	50°S – 45°N	300	66,669	11,890	3.44×10^{10}	4.71×10^{8}
**Total**		1101	359,584	42,050	6.06×10^{10}	9.04×10^{8}

* Note that the number of records for each month varied across grid cells

Supplementary Table 3. Overview of predatory fish taxa recorded in each ocean-specific longline fisheries dataset. Note that a taxon can either represent a single predatory fish species, or a group of (related) predatory fish species (English names of groups of species are given in italics). 'Relative taxon abundance' gives the frequency of each taxon relative to the total catch within an ocean. Taxa with a relative catch greater than 0.1 (10%) of the total catch within an ocean are given in bold (note that the 'OTH' taxon was not excluded in the West Pacific although it was marginally above 10%; the reason for this was that the OTH taxon subsumes several species and the data for the West Pacific would otherwise have become too sparse due to limited taxon resolution, s ee below). Source data are provided in Supplementary Data 1.

Ocean	Taxon abbrev.	Scientific name	English name	Relative taxon abundance
Atlantic	ALB	*Thunnus alalunga*	Albacore	**0.31243**
Atlantic	BET	*Thunnus obesus*	Bigeye tuna	**0.19813**
Atlantic	BFT	*Thunnus thynnus*	Atlantic bluefin tuna	0.05373
Atlantic	BIL	–	*Various marlin and sailfish species not included elsewhere*	0.01038
Atlantic	BLF	*Thunnus atlanticus*	Blackfin tuna	0.00077
Atlantic	BLM	*Makaira indica*	Black marlin	0.00009
Atlantic	BON	*Sarda sarda*	Atlantic bonito	0.00001
Atlantic	BSH	*Prionace glauca*	Blue shark	0.01306
Atlantic	BUM	*Makaira nigricans*	Blue marlin	0.00904
Atlantic	FRI	*Auxis thazard*	Frigate tuna	<0.00001
Atlantic	KGM	*Scomberomorus cavalla*	King mackerel	0.00003
Atlantic	LTA	*Euthynnus alletteratus*	Little tunny	<0.00001
Atlantic	MAK	*Isurus spp*	Mako sharks	0.00016
Atlantic	oSks	–	*Various shark species not included elsewhere*	0.00088
Atlantic	oSmt	–	*Various small tuna species not included elsewhere*	0.00001
Atlantic	oTu	–	*Various tuna species not included elsewhere*	0.00332
Atlantic	POR	*Lamna nasus*	Porbeagle	0.00025
Atlantic	SAI	*Istiophorus albicans*	Atlantic sailfish	0.00494
Atlantic	SBF	*Thunnus maccoyii*	Southern bluefin tuna	0.03031
Atlantic	SKJ	*Katsuwonus pelamis*	Skipjack tuna	0.00072

Atlantic	SMA	*Isurus oxyrinchus*	Shortfin mako	0.00055
Atlantic	SPF	*Tetrapturus pfluegeri*	Longbill spearfish	0.00075
Atlantic	SWO	*Xiphias gladius*	Swordfish	**0.21086**
Atlantic	WAH	*Acanthocybium solandri*	Wahoo	0.00047
Atlantic	WHM	*Tetrapturus albidus*	Atlantic white marlin	0.01246
Atlantic	YFT	*Thunnus albacares*	Yellowfin tuna	**0.13665**
East Pacific	ALB	*Thunnus alalunga*	Albacore	**0.24524**
East Pacific	BET	*Thunnus obesus*	Bigeye tuna	**0.37495**
East Pacific	BIL	–	*Various marlin, sailfish, and spearfish species not included elsewhere*	0.02477
East Pacific	BLM	*Makaira indica*	Black marlin	0.00170
East Pacific	BSH	*Prionace glauca*	Blue shark	0.00610
East Pacific	BUM	*Makaira nigricans*	Blue marlin	0.02197
East Pacific	CCL	*Carcharhinus limbatus*	Blacktip shark	0.00022
East Pacific	FAL	*Carcharhinus falciformis*	Silky shark	0.00895
East Pacific	MAK	–	*Mako sharks not included elsewhere*	0.00023
East Pacific	MLS	*Tetrapturus audax*	Striped marlin	0.07260
East Pacific	OCS	*Carcharhinus longimanus*	Oceanic whitetip shark	0.00022
East Pacific	PBF	*Thunnus orientalis*	Pacific bluefin tuna	0.00031
East Pacific	RSK	–	*Requiem sharks not included elsewhere*	0.00002
East Pacific	SFA	*Istiophorus platypterus*	Indo-Pacific sailfish	0.01238
East Pacific	SKH	–	*Various shark species not included elsewhere; may include: Silvertip shark, Thresher shark, Blacktip reef shark, Copper shark, Bigeye thresher shark, Bignose shark, Bull shark, Galapagos shark, Sandbar shark, Smalltail shark, Night shark, Dusky shark, Longfin mako shark, Pelagic thresher shark, Scoophead, Bonnethead, Great hammerhead, Scalloped hammerhead, Smooth hammerhead, Scalloped bonnethead*	0.00712

East Pacific	SKJ	*Katsuwonus pelamis*	Skipjack tuna	0.00647
East Pacific	SMA	*Isurus oxyrinchus*	Short fin mako shark	0.00053
East Pacific	SPN	–	*Hammerhead sharks not included elsewhere*	0.00003
East Pacific	SSP	*Tetrapturus angustirostris*	Shortbill spearfish	0.00258
East Pacific	SWO	*Xiphias gladius*	Swordfish	0.07942
East Pacific	THR	–	*Thresher sharks not included elsewhere*	0.00026
East Pacific	TUN	–	*Various tuna species not included elsewhere*	0.00070
East Pacific	YFT	*Thunnus albacares*	Yellowfin tuna	**0.13321**
Indian Ocean	ALB	*Thunnus alalunga*	Albacore	**0.29166**
Indian Ocean	BET	*Thunnus obesus*	Bigeye tuna	**0.21434**
Indian Ocean	BLM	*Makaira indica*	Black marlin	0.00664
Indian Ocean	BUM	*Makaira nigricans*	Blue marlin	0.01300
Indian Ocean	MLS	*Tetrapturus audax*	Striped Marlin	0.02312
Indian Ocean	NTAD	–	*Various species not included elsewhere*	0.02043
Indian Ocean	POR	*Lamna nasus*	Porbeagle	0.00010
Indian Ocean	RSK	–	*Requiem sharks not included elsewhere*	0.00006
Indian Ocean	SBF	*Thunnus maccoyii*	Southern bluefin tuna	**0.14683**
Indian Ocean	SFA	*Istiophorus platypterus*	Indo-Pacific sailfish	0.00066
Indian Ocean	SKH	–	*Various shark species not included elsewhere*	0.00674
Indian Ocean	SKJ	*Katsuwonus pelamis*	Skipjack tuna	0.00301
Indian Ocean	SWO	*Xiphias gladius*	Swordfish	0.03952
Indian Ocean	YFT	*Thunnus albacares*	Yellowfin tuna	**0.21642**
West Pacific	ALB	*Thunnus alalunga*	Albacore	**0.36300**
West Pacific	BET	*Thunnus obesus*	Bigeye tuna	**0.19147**
West Pacific	BLM	*Makaira indica*	Black marlin	0.00406
West Pacific	BUM	*Makaira nigricans*	Blue marlin	0.02412
West Pacific	MLS	*Tetrapturus audax*	Striped Marlin	0.02375
West Pacific	OTH	–	*Various species not included elsewhere*	0.10051
West Pacific	SWO	*Xiphias gladius*	Swordfish	0.04435
West Pacific	YFT	*Thunnus albacares*	Yellowfin tuna	**0.24875**

Supplementary Table 4. Summary statistics from Generalized Linear Mixed-effect Models (GAMMs). GAMMs were run with 'latitude' and 'time interval' as predictors of annual relative predation estimates per grid cell. The partial effects of 'latitude' and 'time interval' on relative predation are visualized in Figure 1 and Figure S1A, respectively. As a robustness check, we reran the GAMMs on data restricted to a latitudinal range between -40°S and 40°N, because total pelagic predation at absolute latitudes greater than 40° is likely to be influenced substantially by other predators than by the large pelagic fish predators captured with pelagic longlines (such as marine mammals or demersal fish predators)[1,2]. Note that the results from these spatially-restricted GAMMs were very similar to the GAMMs run on the data from the full latitudinal range. Statistical significance (*P*-value) of the intercept and the partial effects of latitude and time on relative predation was < 0.0001 in all models. See Supplementary Software 1 *(c_Main.Analysis.r)* for the *R*-code.

	**Intercept**	**Adjusted R2 value**	**Intercept**	**Adjusted R2 value**
**Model**	Full latitudinal range (see Table 2)	Full latitudinal range (see Table 2)	Restricted to latitudes 40°S to 40°N	Restricted to latitudes 40°S to 40°N
**West Pacific**	0.020	0.418	0.021	0.413
**East Pacific**	0.019	0.218	0.019	0.257
**Atlantic**	0.020	0.532	0.022	0.574
**Indian Ocean**	0.018	0.600	0.020	0.555

Supplementary Figures

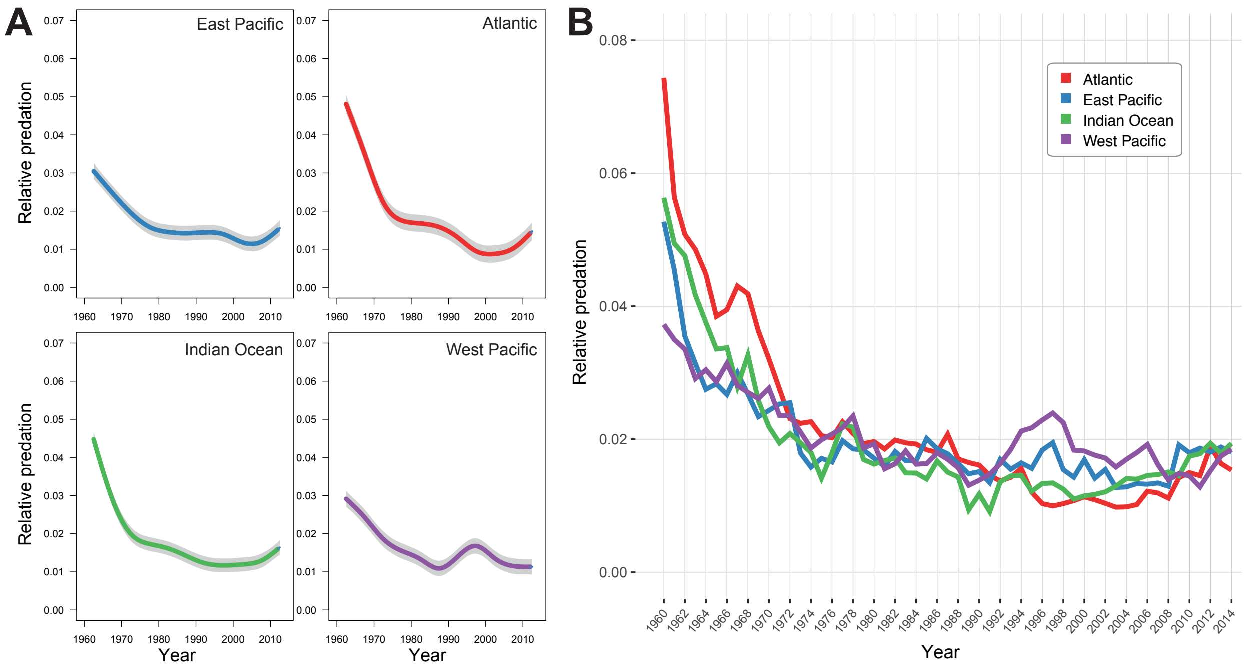

Supplementary Figure 1. Temporal trends in overall relative predation. (A) Partial effect of time ('time interval') on annual relative predation for each ocean basin based on Generalized Additive Mixed-effects Models (see Methods for details). Gray shading indicates the 95% confidence interval. **(B)** Mean annual relative predation per year for each ocean basin. Both (A) and (B) reveal a marked decrease in overall relative predation exerted by pelagic fish predators over time, particularly between 1960 and 1980. Source data are provided in Supplementary Data 1.

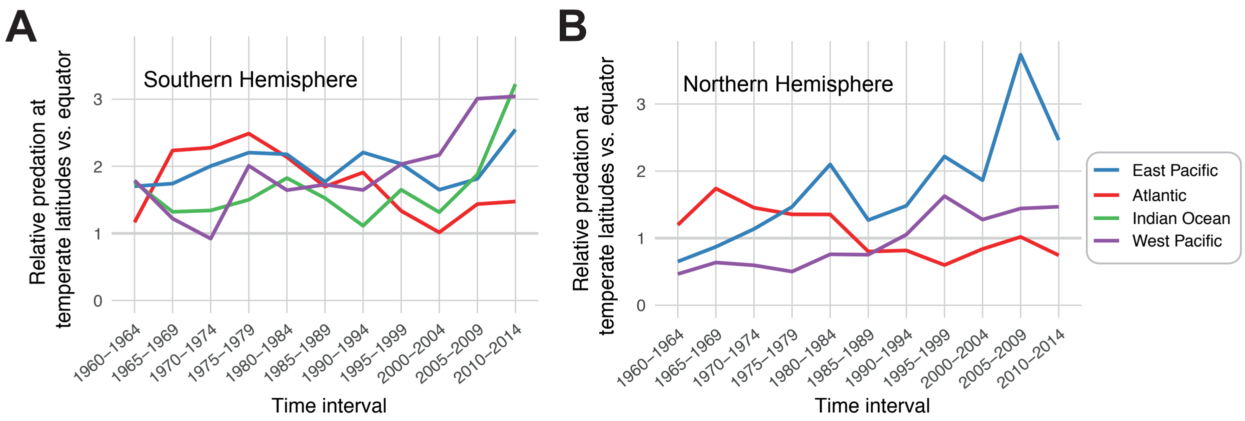

Supplementary Figure 2. Relative predation at temperate latitudes versus the equator across time for the Southern and Northern hemisphere. Shown is the ratio of median annual relative predation at temperate latitudes (30° to 40° on either hemisphere) to the equator (-5°S and 5°N) in five-year time intervals, both for the Southern **(A)** and the Northern **(B)** hemisphere. Values above 1 indicate stronger relative predation in the temperate zone. No data are shown for the Indian Ocean in the Northern Hemisphere because the Indian Ocean extends only marginally beyond 30° in the Northern Hemisphere (see map in Figure 1A). Source data are provided in Supplementary Data 1.

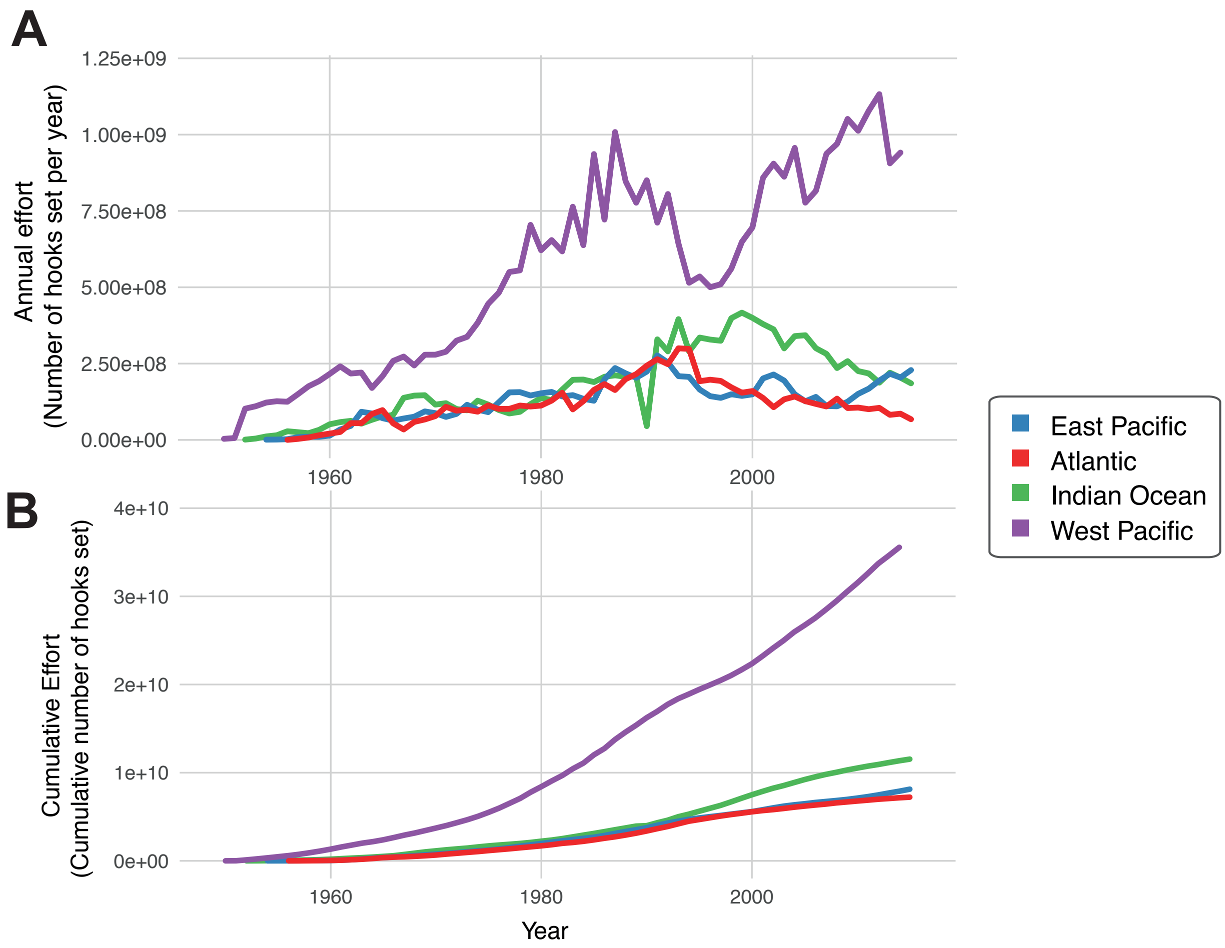

Supplementary Figure 3. Annual and cumulative pelagic longline fishing effort over time. Effort is presented as (A) Annual Effort and (B) Cumulative Effort for each basin. Note the relative minor pelagic longline fishing efforts and catches prior to 1960.

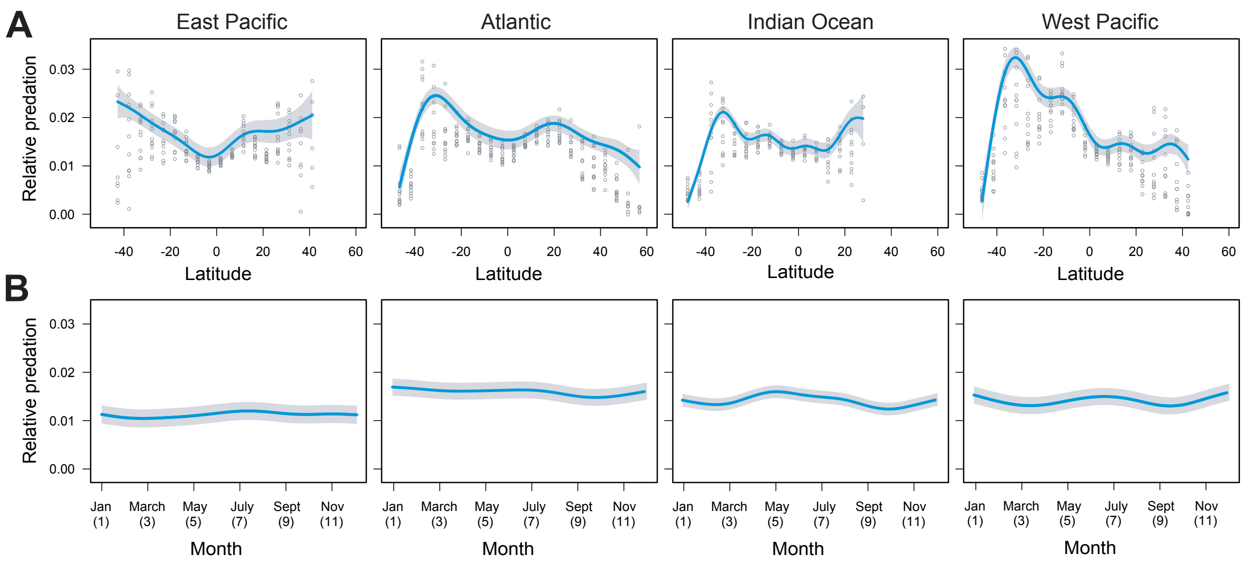

Supplementary Figure 4. Seasonal variation does not influence latitudinal variation in relative predation. For this analysis, annual relative predation data were binned by month and grid cell within five-year time intervals between 1960 and 2014. Thus, instead of working with one relative predation value per grid cell and time interval (as is the case in our default analyses), we here worked with up to 12 values (one per month). The median of all values per every such bin was then taken as input data to run ocean-specific GAMMs that included 'month' (season), besides 'latitude' and 'time interval', to predict relative predation (model formation otherwise equivalent to that of the original GAMMs, see Methods).

(A) Partial effect of 'latitude' on relative predation from these GAMMs. The gray circles represent the median relative predation per month and latitude across all annual relative predation data points between 1960 and 2014 (for visual clarity, a few data points with values >0.03 are not shown). **(B)** Partial effect of 'month' on relative predation from these GAMMs (gray shading indicates the 95% confidence interval).

We conclude that seasonal variation in relative predation is minor (see panel B) and does not influence the overall latitudinal pattern of relative predation (compare panel A to Figure 1B).

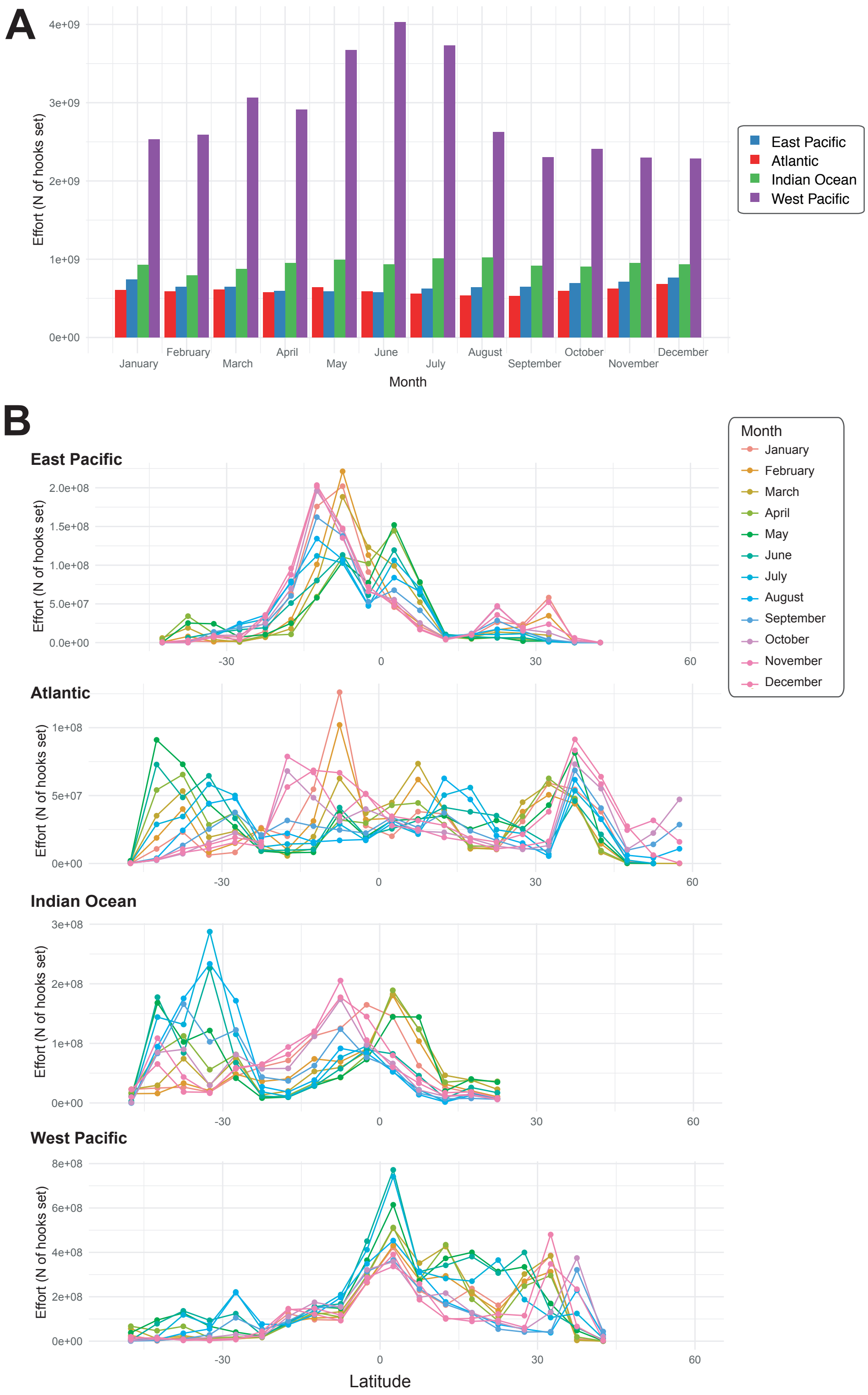

Supplementary Figure 5. Seasonal variation in fishing efforts does not vary consistently across latitude. (A) Total fishing effort per month across all latitudes between 1960 and 2014 shows relatively minor seasonal variation in total fishing

pressure. **(B)** Monthly longline fishing pressure across latitude, considering all catch per effort data from longline fisheries between 1960 and 2014. Despite some seasonal variation in fishing pressure across latitude, this variation does not seem to explain latitudinal patterns of relative predation (see Supplementary Figure 4). Also note that there is a tendency of overall greater fishing pressure close to the equator in the Pacific Ocean. Despite this, increased fishing pressure in unlikely to solely explain the broad-scale latitudinal patterns in relative predation in these two ocean basins (see Supplementary Figures 12 & 13).

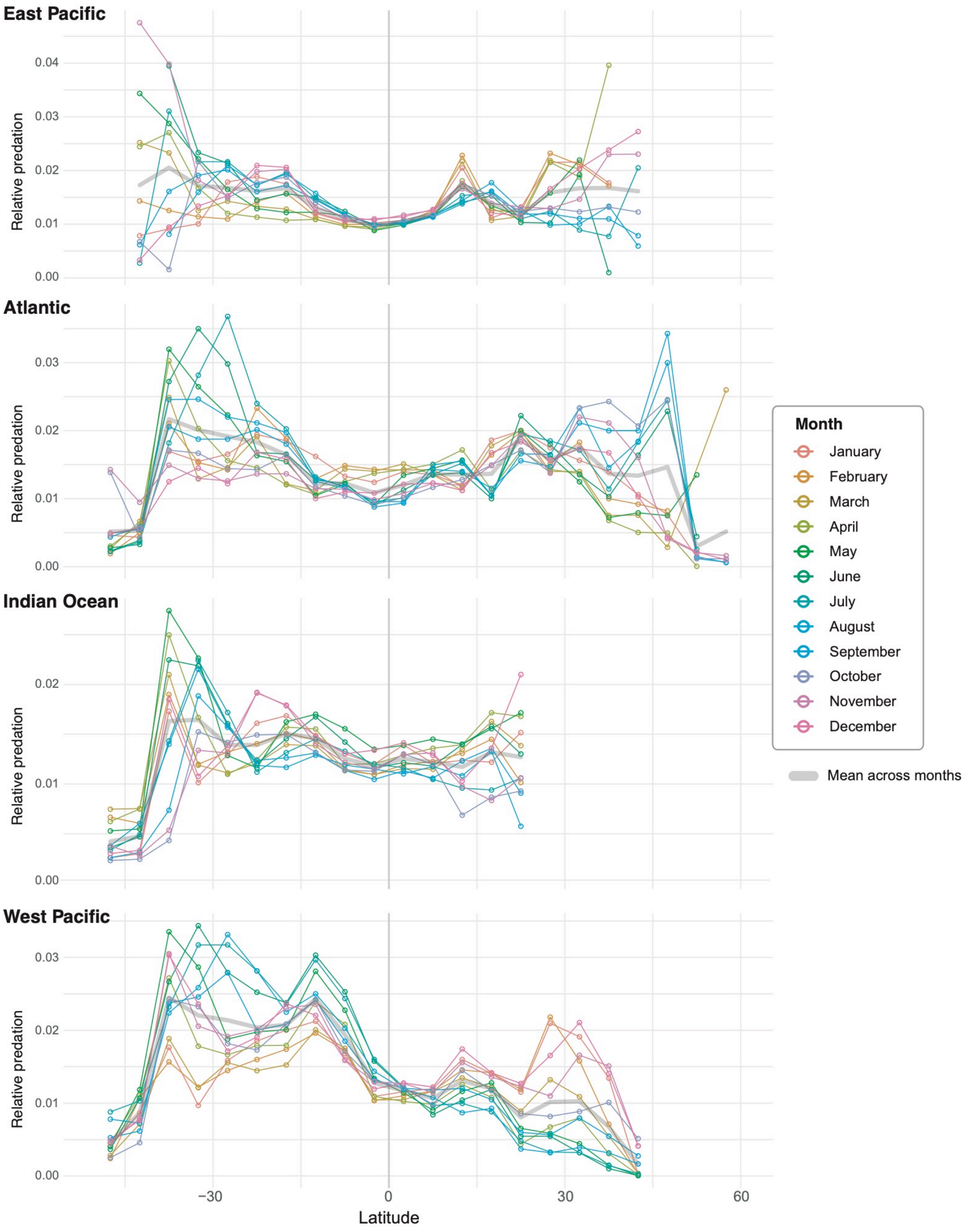

Supplementary Figure 6. Median relative predation per month across latitude considering the full data sets (1960–2014). Seasonal fluctuations in relative predation are stronger at away from the equator. Despite this, the mean across all monthly medians per latitude reveals that the general trend of strongest relative predation at temperate latitudes holds.

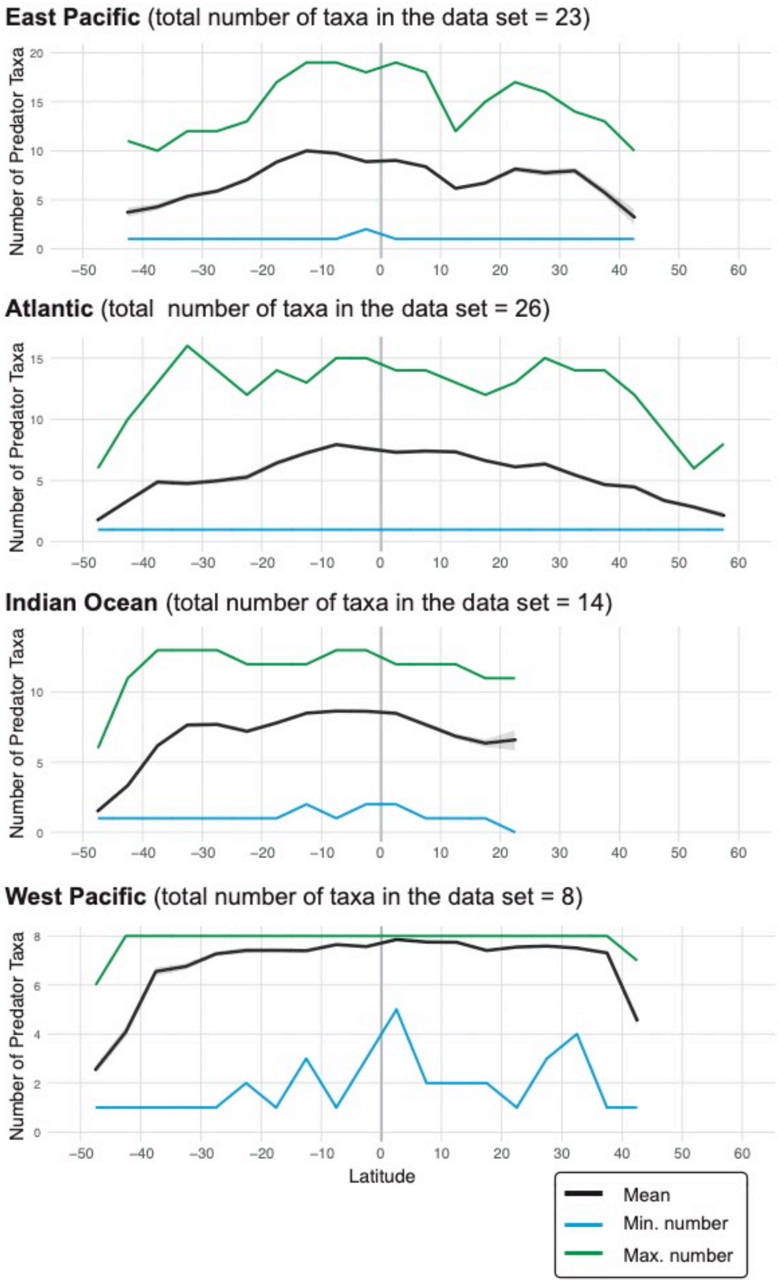

Supplementary Figure 7. The number of reported predatory fish taxa is not higher in temperate regions. Mean (black), minimum (blue) and maximum (green) number of fish predator taxa reported, calculated from presence of fish predators taxon per 5°x5° grid cell within annual catch data. Note that a taxon can either represent a single predatory fish species, or a group of (related) fish predator species (see Supplementary Table 3 for details).

Notably, the number of reported taxa is not higher – but rather lower, if anything – at the latitudes with the strongest relative predation (compare to Figure 1B). Thus, latitudinal differences in reporting standards of predator taxa cannot explain the stronger relative predation at temperate than tropical latitudes. Moreover, the drop in the number of reported predatory fish taxa at latitudes 40° towards the poles corresponds with the general biological shift in the predatory fish community in the ocean from pelagic to benthic species[3]. Finally, we note that several pelagic fish predators largely considered by-catch of logline fisheries (such as many shark species) are rich at temperate latitudes[4], raising the possibility that we may even underestimate the degree to which predation is greater at temperate latitudes than near the equator. Source data are provided in Supplementary Data 1.

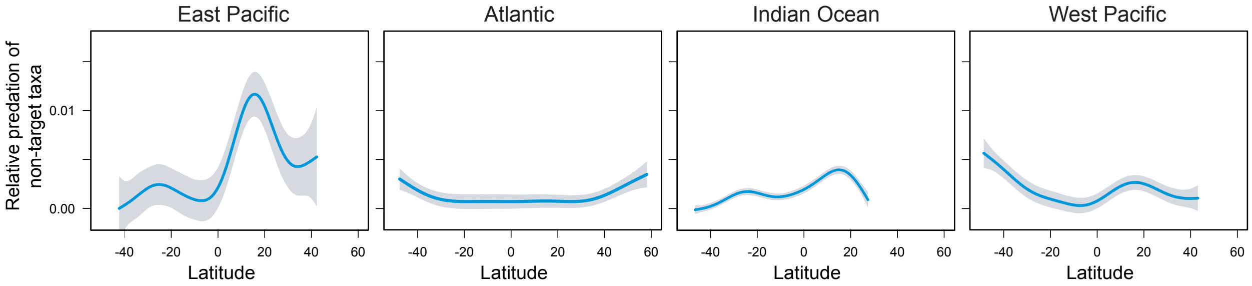

Supplementary Figure 8. Relative predation data excluding the major target species of longline fisheries does not show strongest relative predation at the equator. Fish predators species specifically targeted by longline fisheries are likely to be overrepresented in the total longline catch, and could thus bias the overall latitudinal pattern in predation exerted by large oceanic fish predators. To evaluate whether results based on the main commercially-targeted predatory fish taxa – representing most of the specimens caught by longline fisheries (Supplementary Table 3) – are reflective of general fish predation patterns, we reran our analyses for the subset of data representing only non-major target species. Here, we considered every predator taxon contributing less than 10% to the total catch to be a 'non-target' taxon (Supplementary Table 3). We then used all non-target predator catches to calculate annual 'relative predation of non-target taxa' (one value per grid cell) and ran GAMMs following the same procedure as for the relative predation estimates and GAMM syntax (see Methods for details). Shown is the (partial) effect of 'latitude' on 'non-target predator relative predation' for each ocean, including the 95% confidence interval (gray shading).

In no case does relative predation of non-target fish predators peak at the equator. Observed peaks instead occur at higher, more temperate latitudes, consistent with the results for considering all predatory fish taxa (see Figure 1B). These results suggest that our main conclusions are generally representative of overall patterns of relative predation across latitude and not specific to patterns in commercial fish species. Source data are provided in Supplementary Data 1.

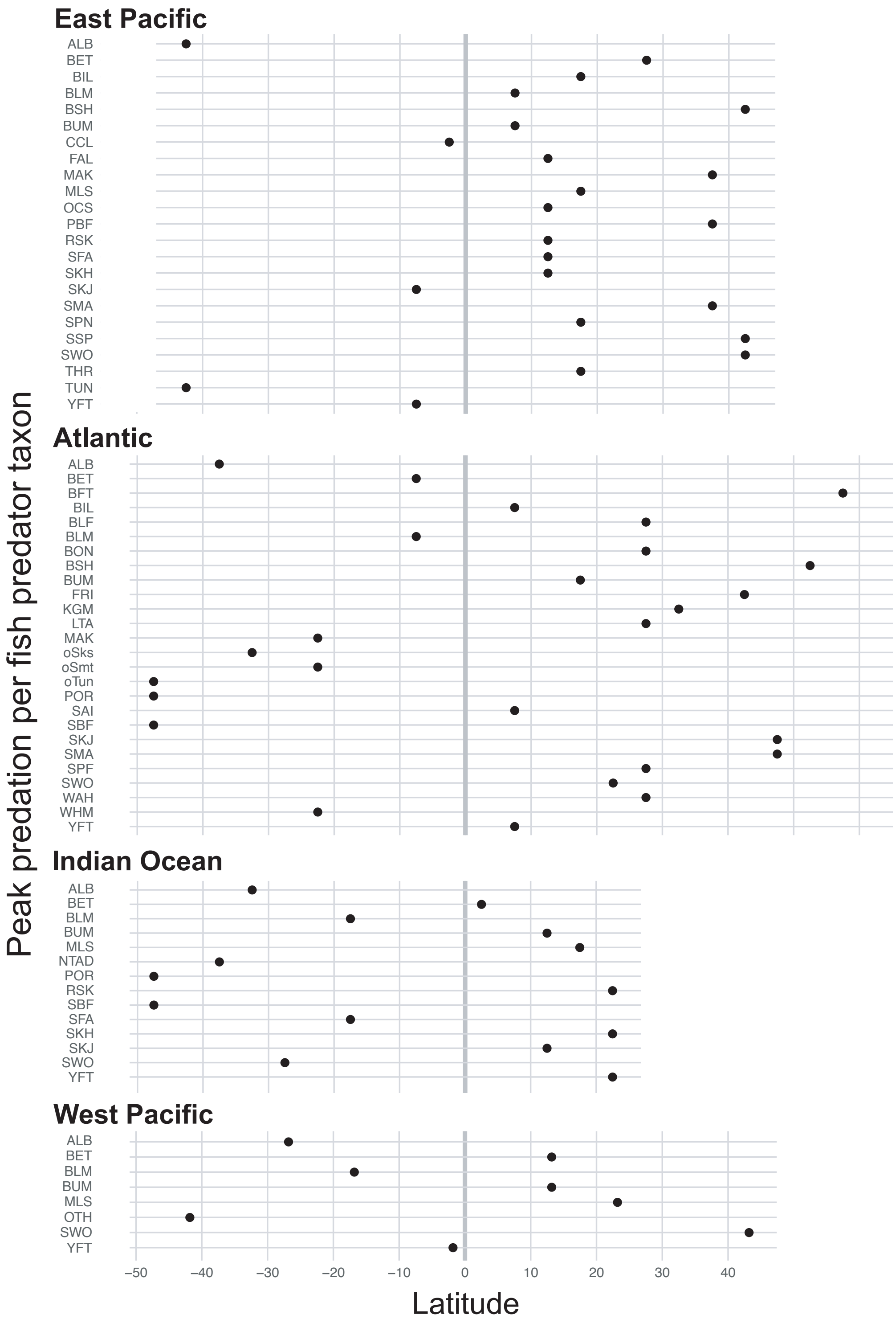

Supplementary Figure 9. Most peaks of taxon-specific predation fall on temperate latitudes, and not on the equator. Latitude with the strongest predation for every predatory fish taxon (for details on taxa names, see Supplementary Table 3). The

predation peak per taxon was inferred based on latitudinal means of annual total catch of every predator taxon, divided by the total effort (nominal CPUE) per grid cell. A summary of this plot is presented as Figure 3.

Some fish predator taxa are likely to be over-/underrepresented in the longline fisheries datasets compared to their actual relative abundance in the entire community of pelagic fish predators (due to being specifically targeted or not by fisheries). Even if this was the case, however, taxon-specific representations across latitude should not be biased by the fact that longline fisheries target certain predator fish species. Importantly, we never find the number of taxon-specific peaks in predation pressure to be highest near the equator (Figure 3). Instead, most peaks fall in all oceans between absolute latitude 20°-30°, supporting our findings of stronger overall relative predation in or near the temperate zone than in the tropics. Source data are provided in Supplementary Data 1.

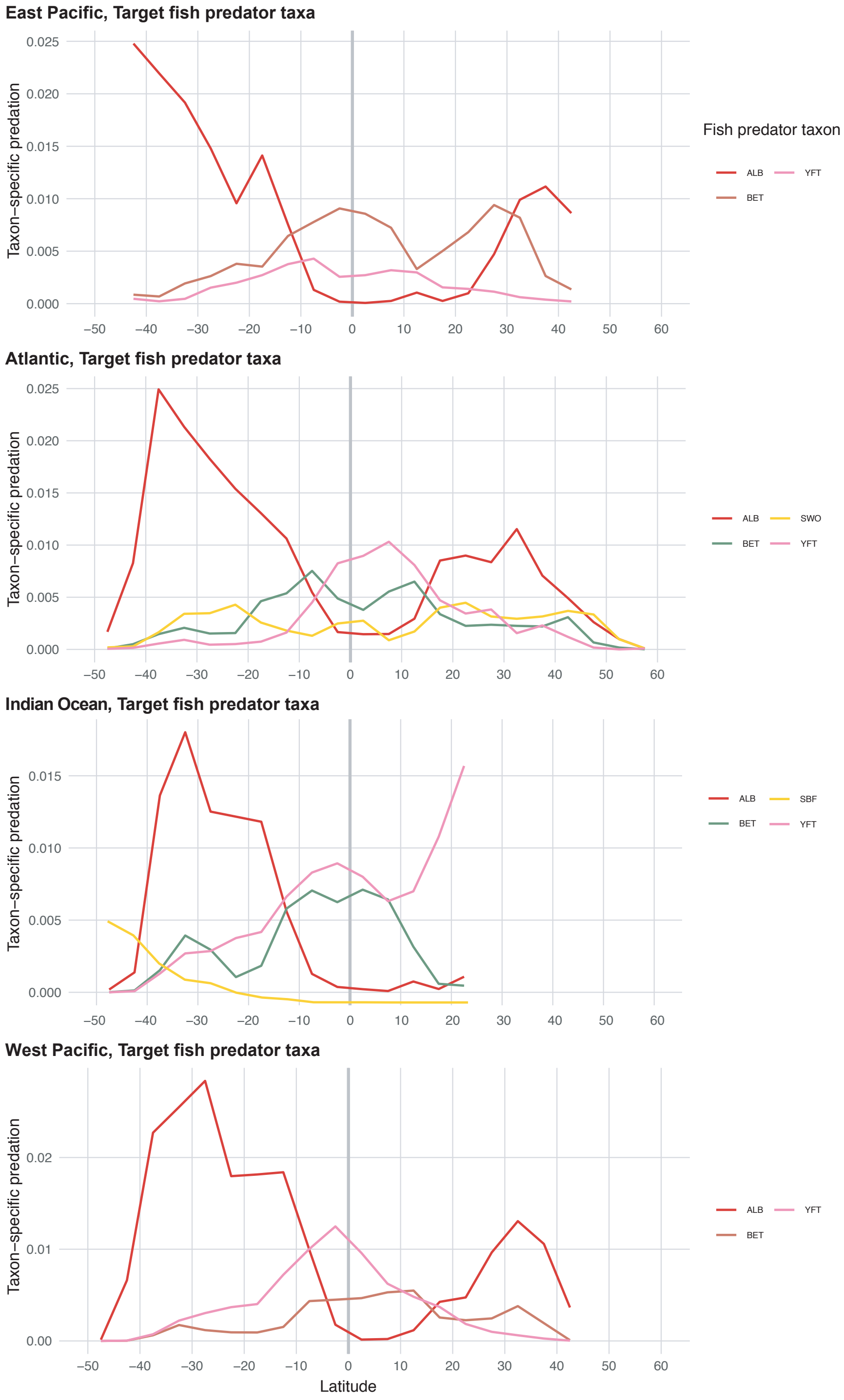

Supplementary Figure 10. Latitudinal patterns of taxon-specific predation for the main target species of longline fisheries. Taxon-specific predation was calculated by the mean per latitude of annual total catch of every predator taxon divided by the summed effort per grid cell. Three letter codes represent individual predatory taxa (Supplementary Table 3). Source data are provided in Supplementary Data 1.

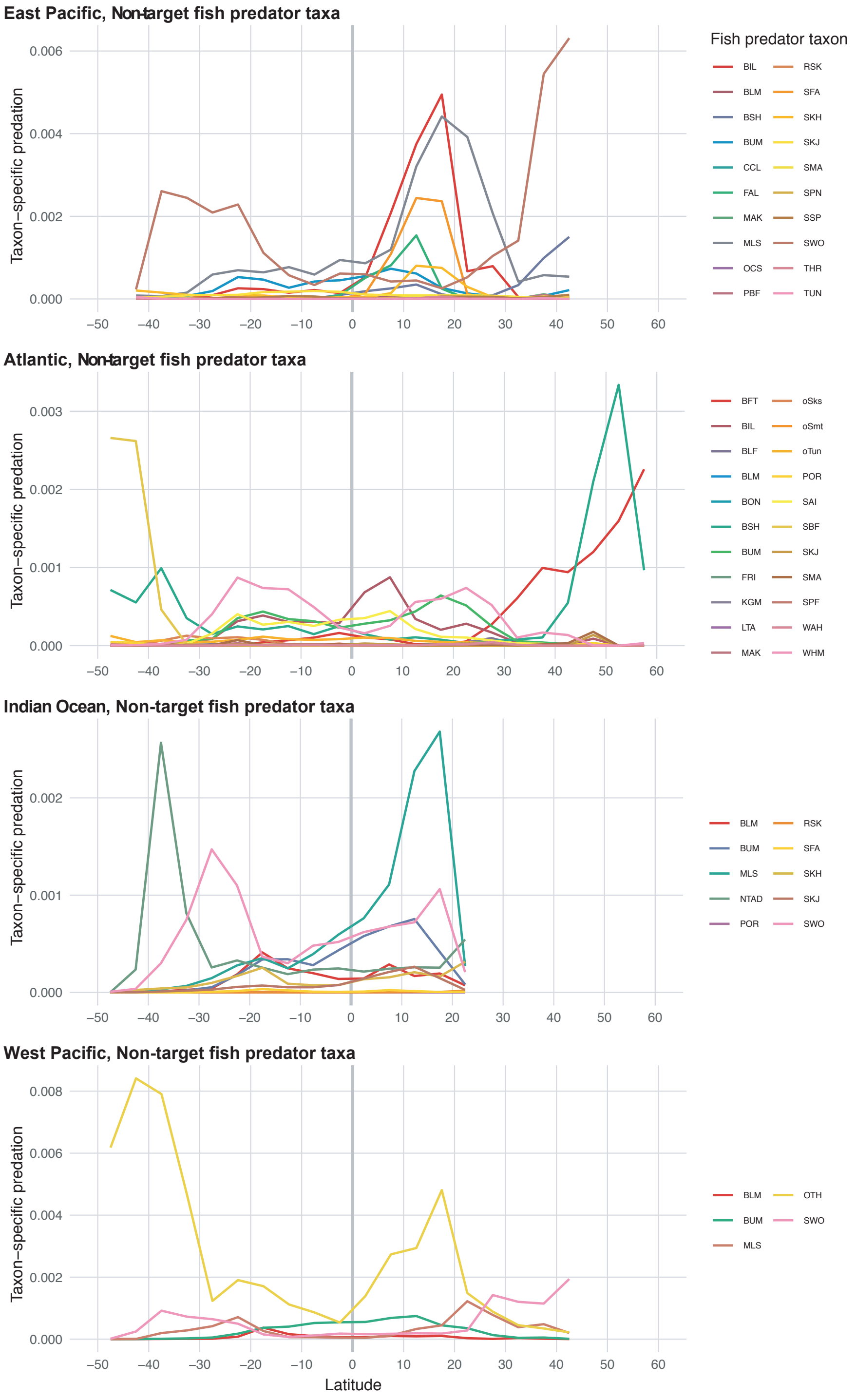

Supplementary Figure 11. Latitudinal patterns of taxon-specific predation for 'non-target taxa' of longline fisheries. Taxon-specific predation was calculated by the mean per latitude of annual total catch of every predator taxon divided by the summed effort per grid cell. Three letter codes represent individual predatory taxa (Supplementary Table 3). Source data are provided in Supplementary Data 1.

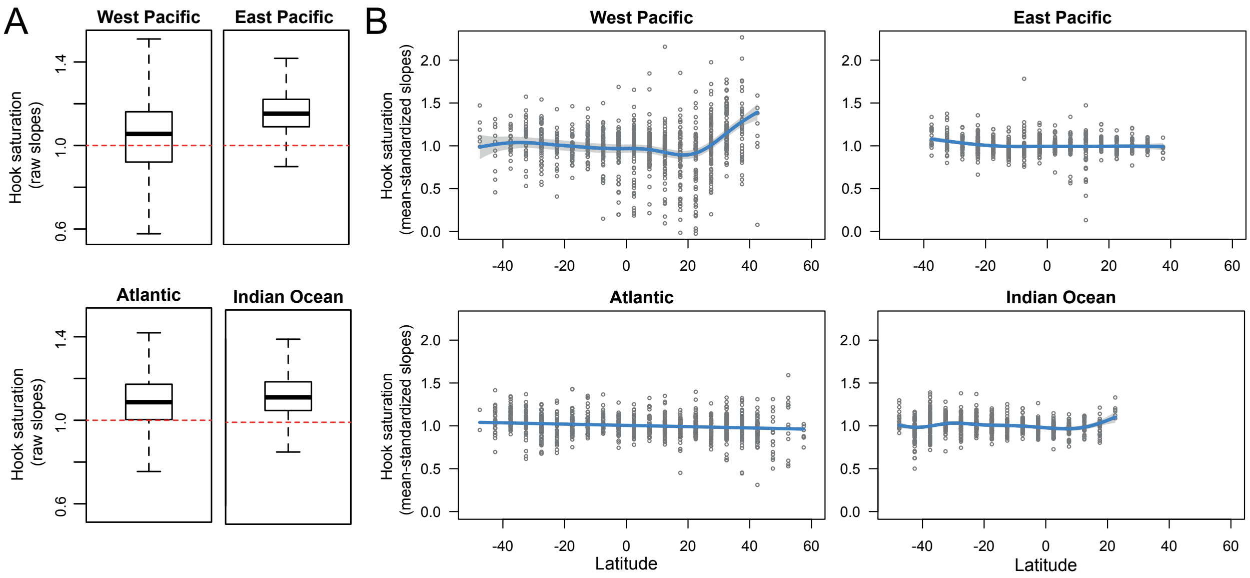

Supplementary Figure 12. Uneven hook saturation is unlikely to bias latitudinal patterns of variation in relative predation. **(A)** Shown are boxplots of all raw slope values from exponential models relating catch to effort (see Methods for further details). Slopes are generally above 1, indicating that saturation does not influence estimates of relative predation on average. Box plots represent median and IQR values, whiskers extend to 1.5 times the IQR. **(B)** Partial effects of latitude on slope based on the GAMMs. Blue lines represent the estimated GAMM function with grey shading indicating 95% confidence intervals. Hook saturation is relatively even across the majority of latitudes for most oceans, although there is a marked increase in slope from latitude 20° onwards in the Northern hemisphere in the West Pacific. However, the West Pacific shows peaks in relative predation at temperate latitudes in both hemispheres, with the highest rates at temperate southern latitudes. Thus any potential issues with saturation in this ocean care unlikely to explain the patterns in relative predation observed (Figure 1). Source data are provided in Supplementary Data 1.

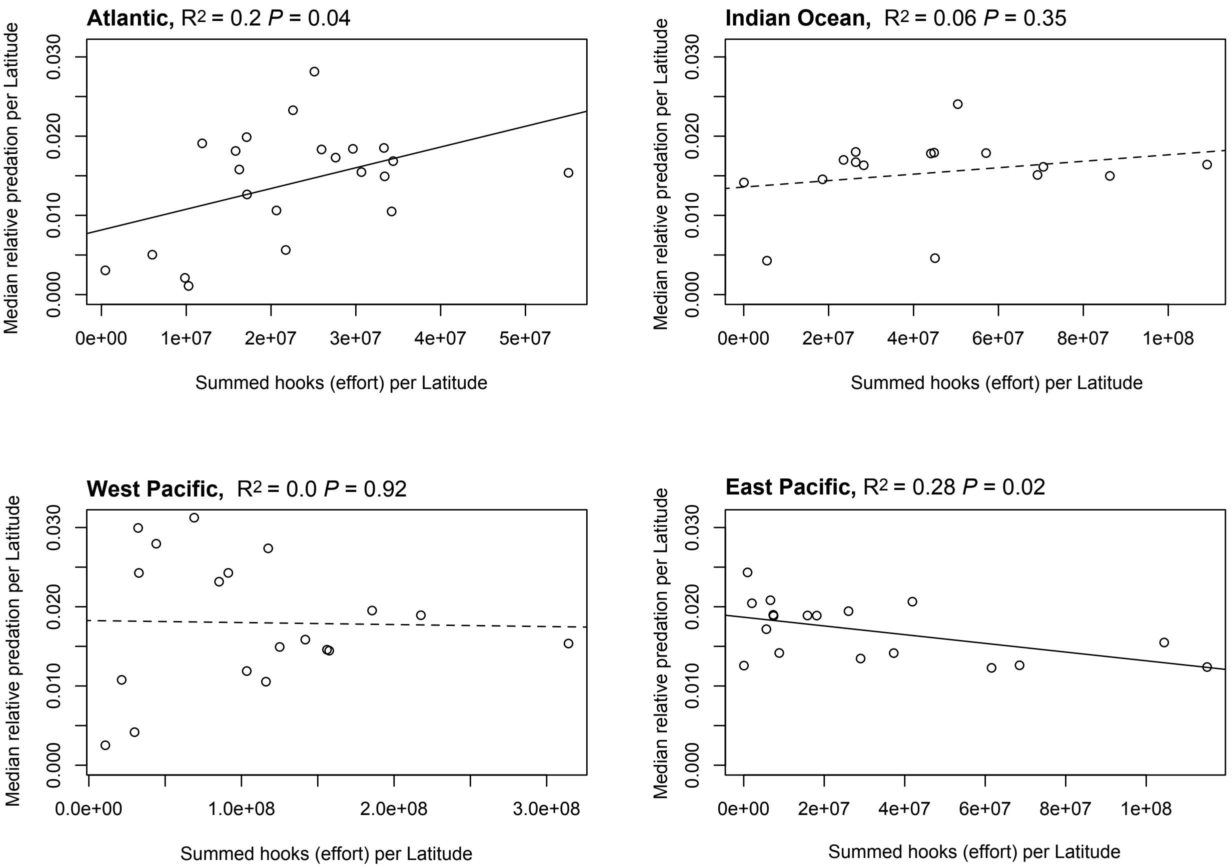

Supplementary Figure 13. Test for a relationship between the total number of hooks set and the median relative predation across latitude. Association between the total number of hooks set (effort) and the median annual relative predation across latitudes between 1960 and 2014. Because oceans differ in their longitudinal extent at different latitudes, the total effort per latitude was standardized by the longitudinal extend (i.e., the number of 5° x 5° grid cells) of that latitude. Despite weak associations between the overall relative predation and effort in the Atlantic and East Pacific, these associations differ in direction and are unlikely to explain the consistent latitudinal pattern in relative predation across oceans (Figure 1B). Moreover, we note that the statistically positive association in the Atlantic is the consequence of geographically extended – yet minor – fishing effort at latitudes beyond 40° toward the poles (see Figure 1), where pelagic fish predators are known to be rare[5]. After excluding latitudes beyond 40° polewards, the R^2 drops to 0.07 (P = 0.32) for the Atlantic. P-values denote the statistical significance of the effect of effort (summer number of hooks per latitude) on predation (median relative predation per latitude) in a linear regression. Source data are provided in Supplementary Data 1.

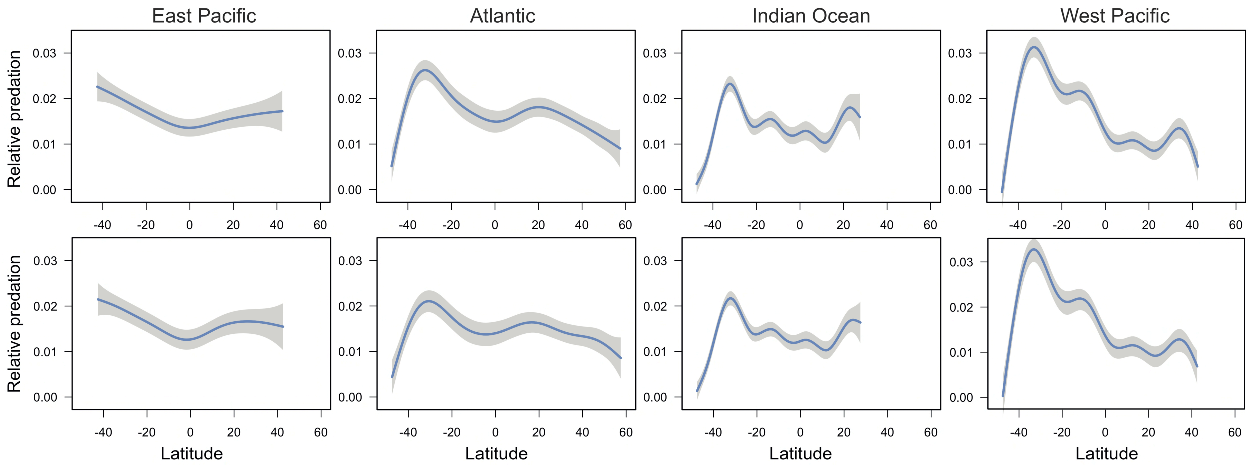

Supplementary Figure 14. Effect of extreme values ('outliers') on latitudinal patterns of relative predation. The (partial) effect of latitude on annual relative predation in GAMMs with extreme values removed (top; depicted GAMM functions correspond to the ones in Figure 1B) and with extreme values included (bottom). Outliers influence the latitudinal relative predation patterns only marginally. Gray shading indicates the 95% confidence interval.

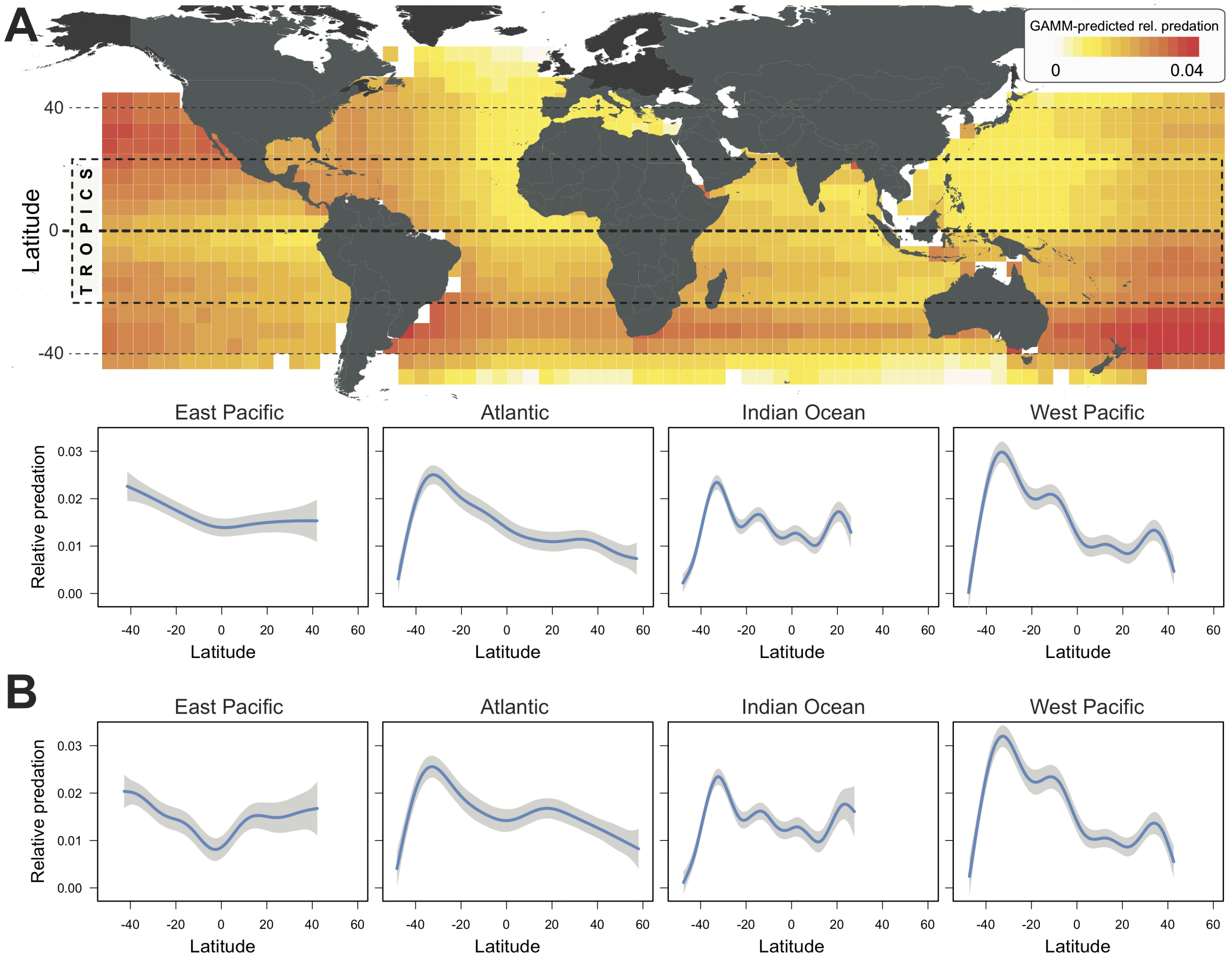

Supplementary Figure 15. Accounting for other aspects of space does not change patterns of latitudinal variation in relative predation. (A) Global map of GAMM-predicted relative predation using 'longitude' besides 'latitude' and 'time interval' to relative predation (model formation otherwise equivalent to that of the original GAMMs, see Methods in main text). Below the map, the partial effect of 'latitude' from these GAMMs is shown. **(B)** Partial effect of 'latitude' in a GAMM that also included 'minimum distance to land' and 'ocean depth' besides 'time interval' and 'latitude' to predict relative predation (all else being equal to the default GAMMs). GAMMs in both (A) and (B) were based on the median annual relative predation per five-year time interval and 5°x5° grid cell (see Methods for further details). Gray shading indicates the 95% confidence interval.

'Minimum distance to land' was calculated using the *gDistance* function in the *rgeos* package in *R* after converting grid cell midpoints and standard world map shapefiles to equidistance projections. Landmasses smaller than 10 polygons were not

considered. Custom scripts are available as Supplementary Software 1 *(c_Distance.to.land.r)*.

The dataset "GEBCO_BATHY_2002-01-01_rgb_360x180.SS" retrieved from *https://neo.sci.gsfc.nasa.gov/view.php?datasetId=GEBCO_BATHY* was used to calculate the mean 'ocean depth' per 5°x5° grid cell from ocean depth data at a 1°x1° resolution. Using instead of the mean the *maximum* or *minimum* ocean depth per grid cell yielded highly similar results. Custom scripts are available as Supplementary Software 1 (*c_Ocean.depth.r*).

We conclude that although some variation in relative predation is explained by spatial variables beyond latitude – such as by longitude (see A), latitudinal patterns of relative predation remain largely unchanged when accounting for these other spatial variables (Figure 1B). As well, we observe increased predation along some coast lines as might be expected due to increased productivity along continental shelves, but when distance from shore is included in our models, the results are very similar to our main analyses (Figure 1). Source data are provided in Supplementary Data 1.

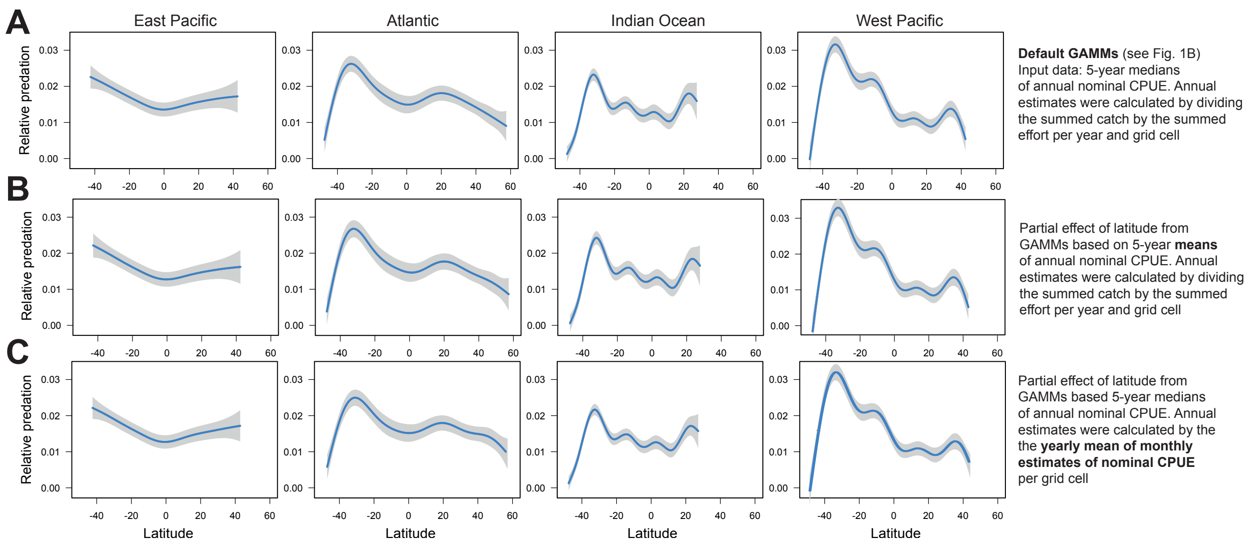

Supplementary Figure 16. Partial effect of 'latitude' on relative predation when using alternative ways of calculating nominal CPUE per five-year time interval and grid cell. (A) (Partial) effect of 'latitude' on relative predation from GAMMs ran at default (see Methods; Figure 1B). Gray shading indicates the 95% confidence interval. **(B)** (Partial) effect of 'latitude' on relative predation using the default GAMM syntax, but the input data are means – and not medians – of annual estimates per five-year time interval and grid cell. **(C)** (Partial) effect of 'latitude' on relative predation using the default GAMM syntax, but with the input data being five-year medians of annual relative predation estimates calculated from the mean – and not the summed – nominal CPUE across all monthly records per grid cell. In each of these cases, the latitudinal patterns of relative predation are highly similar.

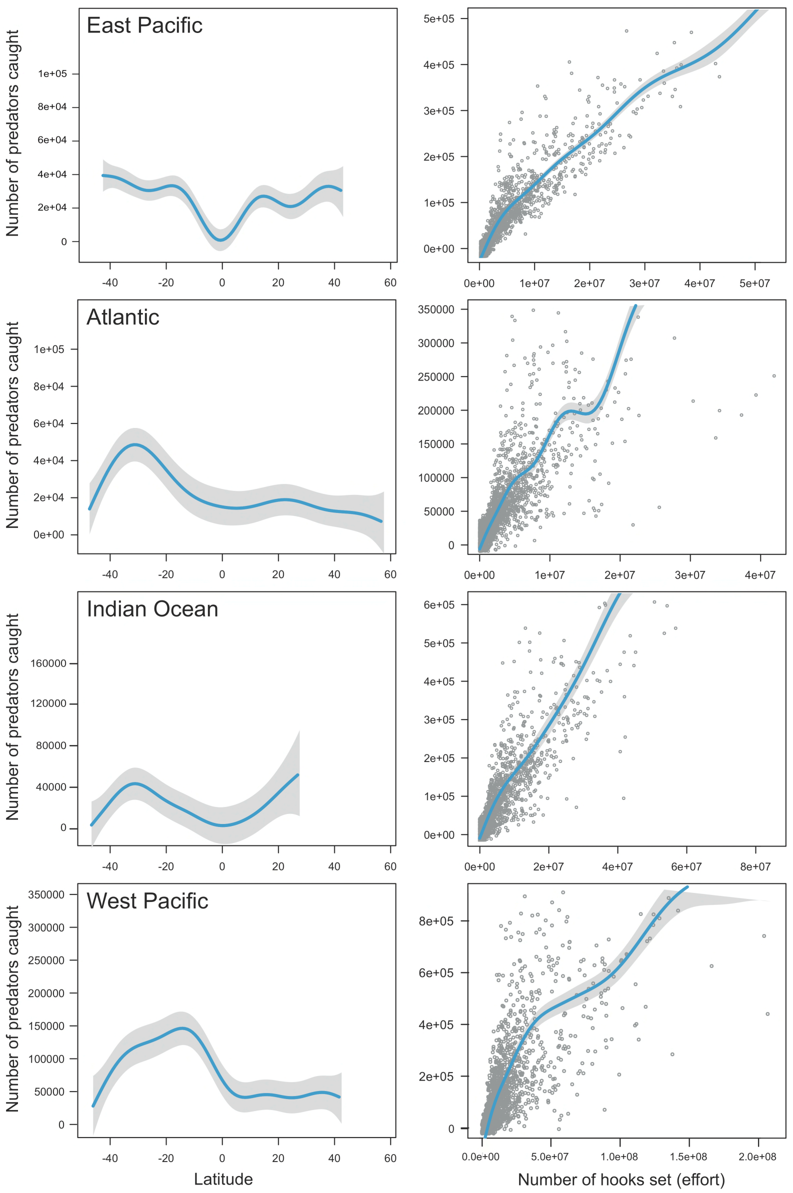

Supplementary Figure 17. An alternative approach to infer relative predation by large fish predators reveals similar latitudinal predation patterns to the ones reported from the default approach. We used annual sums of total catch and total effort per grid cell as data points to run GAMMs with 'latitude', 'time' (year), and 'effort' (number of hooks set) as fixed effects, and 'catch' (number of fish predators caught) as explanatory variable. Shown are the partial effects of 'latitude' (left panels) and that of 'effort' (right panels; for clarity, some data points with a very high number of catch are not shown) on the number of predators caught. Gray shading indicates the 95% confidence interval. Results from these GAMMs support the notion from the other analyses of a greater relative predation at temperate latitudes than at the equator.

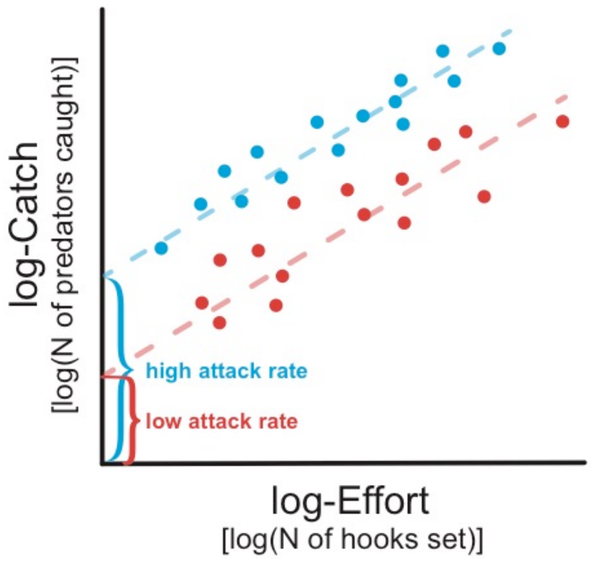

Supplementary Figure 18. Alternative calculation of relative predation based on the intercept of the relationship between catch and effort across all records in a given location (grid cell) and time period. Locations with relatively more catch per unit effort across individual records (blue) will result in a larger intercept in a linear model of log-catch as a function of log-effort than locations with less catch per effect across individual records (red).

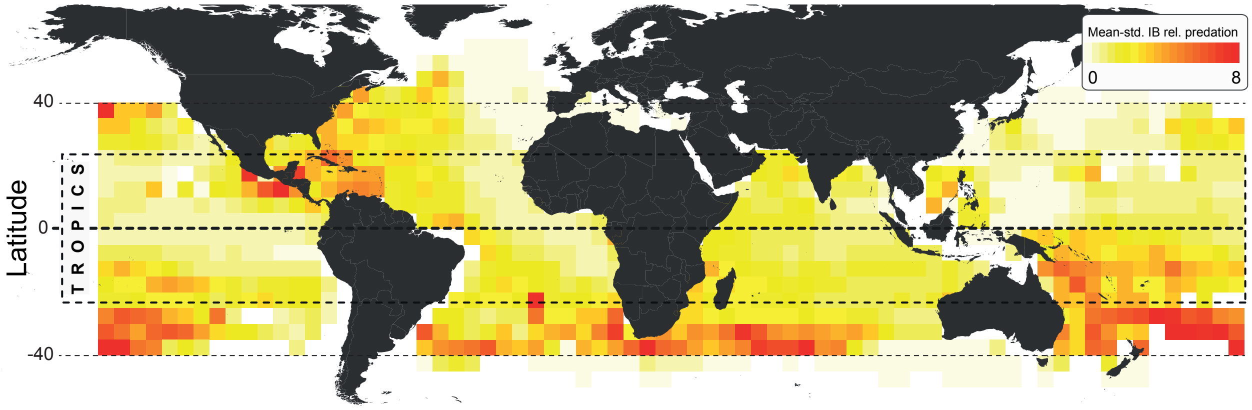

Supplementary Figure 19. Global distribution of intercept-based (IB) relative predation. Colours indicate average IB relative predation (after standardizing each value by the mean of all values in a given time period to account for the effect of time, see Supplementary Figure 21) per 5° x 5° grid cell. As per patterns based on annual relative predation (Figure 1A), IB relative predation is stronger in the temperate zone than in the tropics, especially in the Southern Hemisphere. Source data are provided in Supplementary Data 1.

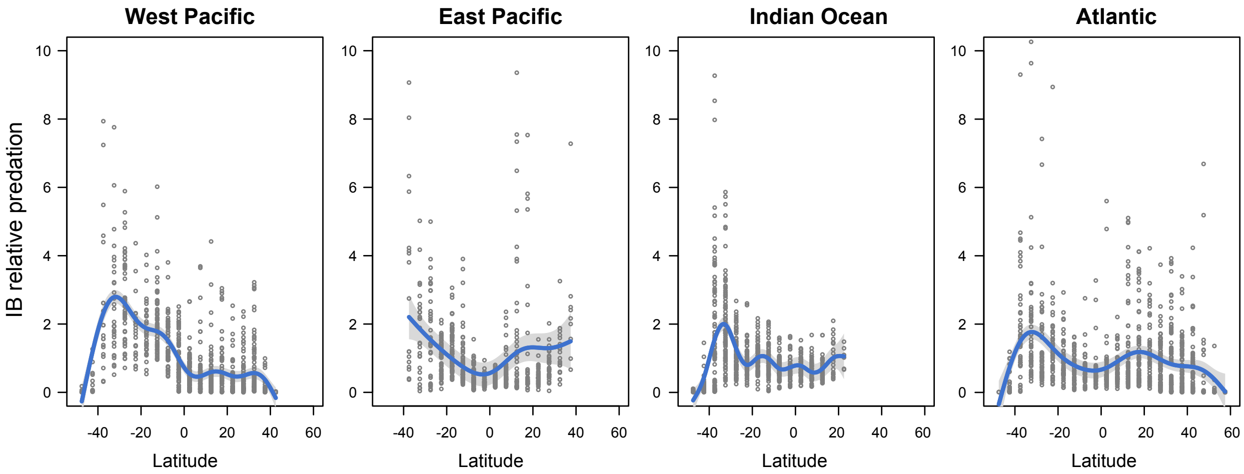

Supplementary Figure 20. Latitudinal variation in intercept-based (IB) relative predation. IB relative predation was estimated per 11-year time period, with five time points overall (i.e., 1960-1970, 1971-1981, ..., 2004-2014). GAMMs that included 'time period' as fixed effect (akin to 'time interval' in the GAMMs presented in the main text) did not converge. We thus first standardized IB relative predation by the mean IB relative predation across all cells for each representative time period to account for the effect of time in the data (see Supplementary Figure 21), and ran GAMMs with 'latitude' as a fixed effect, 'GridID' as random variable, and by accounting for spatial autocorrelation (see Methods for details). Blue lines represent the estimated GAMM function with grey shading indicating 95% confidence intervals. Grey dots show IB relative predation at each latitude (standardized by the mean per time slice in B; for clarity, a few values greater than 10 are not shown). The statistical significance of the effect of latitude on IB relative predation was < 0.001 in all GAMMs. Source data are provided in Supplementary Data 1.

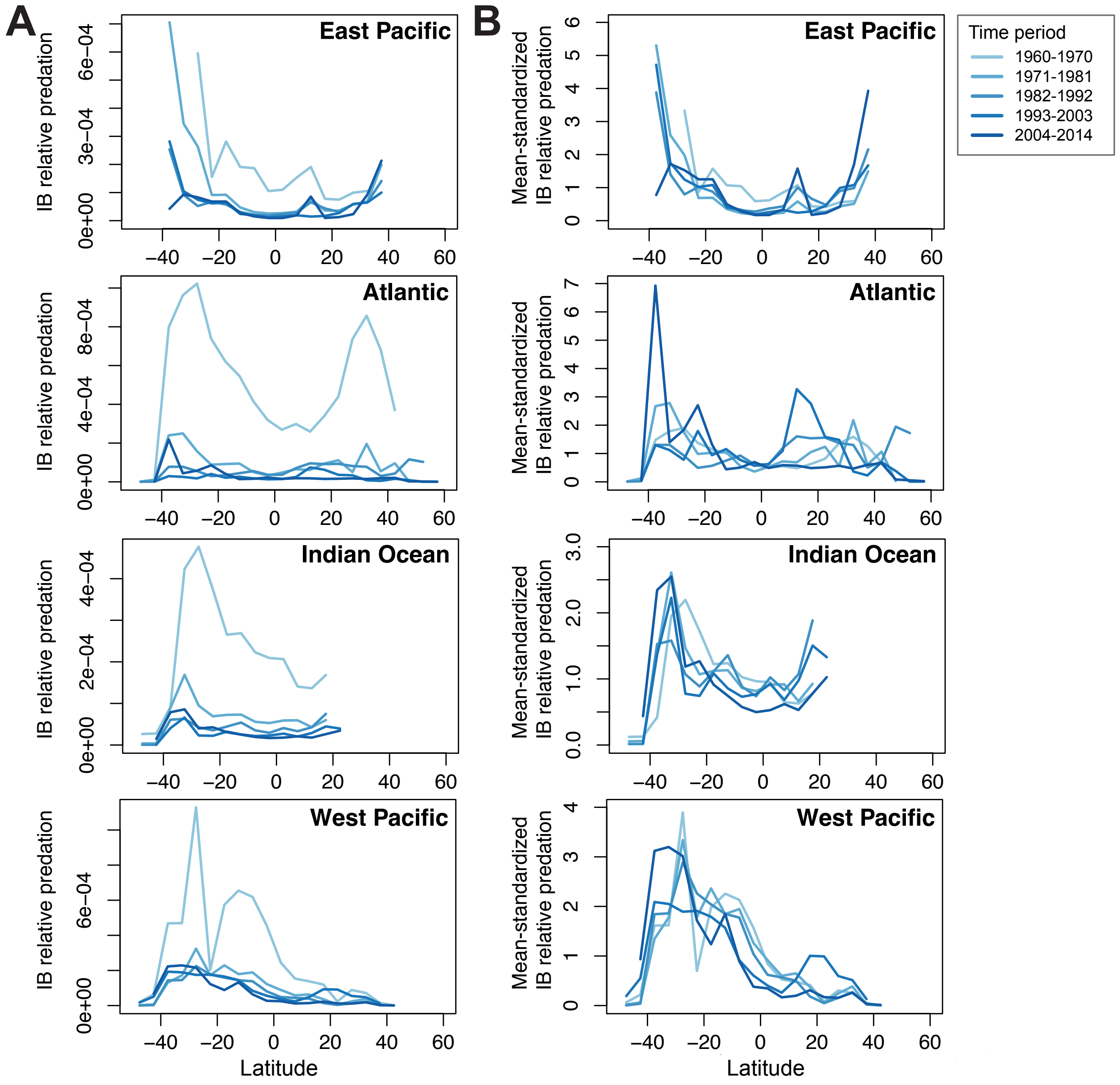
A
B
East Pacific
Atlantic
Indian Ocean
West Pacific
IB relative predation
Mean-standardized IB relative predation
Latitude
Time period
1960-1970
1971-1981
1982-1992
1993-2003
2004-2014

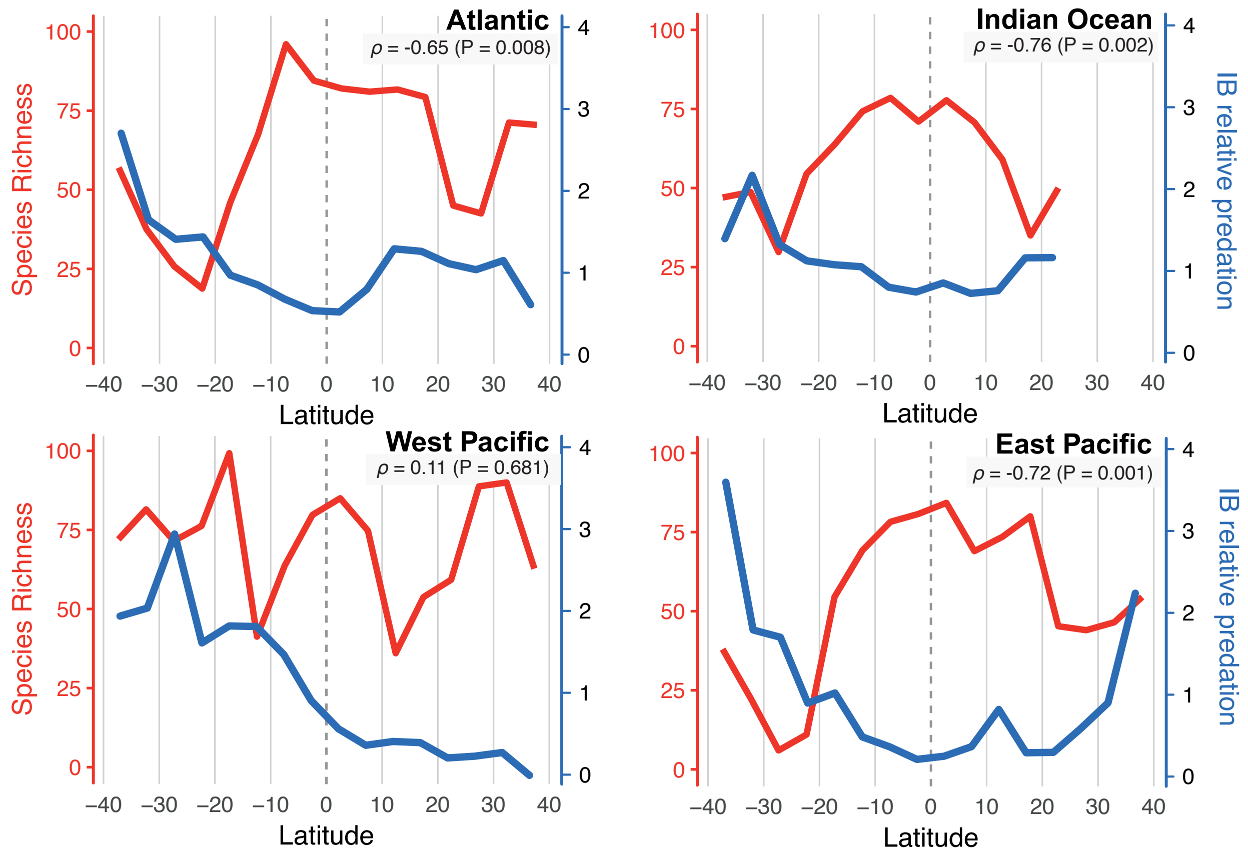

Supplementary Figure 22. The relationship between intercept-based (IB) relative predation and species richness across latitude for each ocean basin. Shown is median IB relative predation after mean-standardization to remove the effect of time (Supplementary Figure 21). Consistent with results from analyzing annual relative predation (Figure 4), IB relative predation shows a negative association with species richness across latitude. Spearman's rho and the statistical significance (non-parametric *P*-values) of the association between median species richness and median IB relative predation across latitude are given in the top right of each plot. Source data are provided in Supplementary Data 1.

Supplementary References

1 Meyer, J. R. & Kassen, R. *Nature* **446**, 432 (2007).
2 Arbuckle, K. & Speed, M. P. *Proc. Natl. Acad. Sci. USA* **112**, 13597-13602 (2015).
3 van Denderen, P. D. *et al. Nat. Ecol. Evol.* **2**, 65-70 (2018).
4 Lucifora, L. O. *et al. PLoS One* **6**, e19356 (2011).
5 Nosil, P. & Crespi, B. J. *Proc. Natl. Acad. Sci. USA* **103**, 9090-9095 (2006).
