# Supplementary material for: Pelagic fish predation is stronger at temperate latitudes than near the equator
Source: Nat Commun. 2020 Mar 31;11:1527. doi: 10.1038/s41467-020-15335-4 (PMC7109113; doi:10.1038/s41467-020-15335-4)
Supplement: Supplementary file 3 — Reporting Summary [file 41467_2020_15335_MOESM3_ESM.pdf]

nature research

Corresponding author(s): Marius Roesti
Last updated by author(s): Feb 18, 2020

Reporting Summary

Nature Research wishes to improve the reproducibility of the work that we publish. This form provides structure for consistency and transparency in reporting. For further information on Nature Research policies, seeAuthors & Referees and theEditorial Policy Checklist .

Statistics

For all statistical analyses, confirm that the following items are present in the figure legend, table legend, main text, or Methods section.

n/a	Confirmed	
☐	☒	The exact sample size (*n*) for each experimental group/condition, given as a discrete number and unit of measurement
☐	☒	A statement on whether measurements were taken from distinct samples or whether the same sample was measured repeatedly
☐	☒	The statistical test(s) used AND whether they are one- or two-sided<br>*Only common tests should be described solely by name; describe more complex techniques in the Methods section.*
☐	☒	A description of all covariates tested
☐	☒	A description of any assumptions or corrections, such as tests of normality and adjustment for multiple comparisons
☐	☒	A full description of the statistical parameters including central tendency (e.g. means) or other basic estimates (e.g. regression coefficient) AND variation (e.g. standard deviation) or associated estimates of uncertainty (e.g. confidence intervals)
☐	☒	For null hypothesis testing, the test statistic (e.g. *F*, *t*, *r*) with confidence intervals, effect sizes, degrees of freedom and *P* value noted<br>*Give P values as exact values whenever suitable.*
☒	☐	For Bayesian analysis, information on the choice of priors and Markov chain Monte Carlo settings
☒	☐	For hierarchical and complex designs, identification of the appropriate level for tests and full reporting of outcomes
☐	☒	Estimates of effect sizes (e.g. Cohen's *d*, Pearson's *r*), indicating how they were calculated

Our web collection on statistics for biologists contains articles on many of the points above.

Software and code

Policy information about availability of computer code

Data collection	No data collection software was used. All data analyzed are publicly available. Please see Table S1 and Aquamaps for details
Data analysis	R scripts used to process data (data filtering), and to obtain and visualize main study results are made available as part of the Supplementary Material. Data analyses and visualization were conducted in R (R version 3.5). R-packages (and their version) used are:<br>adehabitatHR (0.4.16)<br>foreign (0.8.72)<br>ggplot2 (3.2.1)<br>Hmisc (4.3.0)<br>mapdata (2.3.0)<br>maps (3.3.0)<br>maptools (0.9.9)<br>mgcv (1.8.31)<br>raster (3.0.7)<br>rgdal (1.4.8)<br>rgeos (0.5.2)<br>rmapshaper (0.4.1)<br>stringr (1.4.0)<br>visreg (2.6.0)

For manuscripts utilizing custom algorithms or software that are central to the research but not yet described in published literature, software must be made available to editors/reviewers. We strongly encourage code deposition in a community repository (e.g. GitHub). See the Nature Research guidelines for submitting code & software for further information.

Data

Policy information about availability of data

All manuscripts must include a data availability statement. This statement should provide the following information, where applicable:

- Accession codes, unique identifiers, or web links for publicly available datasets
- A list of figures that have associated raw data
- A description of any restrictions on data availability

All data analyzed in this study are publicly available, please see Table S1 and Aquamaps (https://www.aquamaps.org/) for details.

Field-specific reporting

Please select the one below that is the best fit for your research. If you are not sure, read the appropriate sections before making your selection.

☐ Life sciences ☐ Behavioural & social sciences ☒ Ecological, evolutionary & environmental sciences

For a reference copy of the document with all sections, see nature.com/documents/nr-reporting-summary-flat.pdf

Ecological, evolutionary & environmental sciences study design

All studies must disclose on these points even when the disclosure is negative.

Study description	This study investigates latitudinal patterns of fish predation in the open ocean. Longline fisheries catch-per-unit-effort data from the world's major oceans (West & East Pacific, Indian Ocean, Atlantic) collected between 1960 and 2014 were analyzed. Data were analyzed using multivariate statistics (GAMMs), while trying to account for both spatial and temporal autocorrelation.
Research sample	We investigated patterns of predation that is exerted by large pelagic fish predators based on four publicly available datasets from pelagic longline fisheries (West Pacific, East Pacific, Atlantic and Indian Ocean), each managed by an independent commission. Combined, these data span all of the world's major oceans. Source links of these data sets are:<br>Atlantic: https://www.iccat.int/Data/t2ce_20171120.zip<br>East Pacific : http://www.iattc.org/PublicDomainData/IATTC-Catch-by-species1.htm<br>West Pacific: https://www.wcpfc.int/wcpfc-public-domain-aggregated-catcheffort-data-download-page<br>Indian Ocean: http://www.iotc.org/documents/ce-reference<br>All datasets included catch-per-effort records per month and year and were generally at spatial resolutions of 1°x1° or 5°x5° latitudinal grids. A small percentage of the records was reported at lower resolution and was removed. For consistency, we aggregated data presented at 1°x1° resolution to the lower resolution by assigning records to the nearest geographic midpoint of an overlaid 5°x5° grid. Overall, these data allow us to conduct a global test of the strength of pelagic fish predation in the ocean.
Sampling strategy	No tests to determine sample sizes were done, because pre-existing (given) data were analyzed. We note that the data were extensive, with over 350,000 individual data records post data quality filtering (see Table S2 for details).
Data collection	Data are publicly available and contain monthly catch-per-effort data records from longline fisheries. Data are curated by independent commissions: "International Commission for the Conservation of Atlantic Tunas" (ICCAT; Atlantic), "Inter-American Tropical Tuna Commission" (IATTC; East Pacific), "Western and Central Pacific Fisheries Commission" (WCPFC; Western & Central Pacific), "Indian Ocean Tuna Commission" (IOTC; Indian Ocean). Source links are given in Table S1.
Timing and spatial scale	Data analyzed span the years 1960-2014 and span a maximal latitudinal range of 50°S to 60°N. Raw data are aggregated by month, year, and grid cell. Details can be obtained from Table S1 and S2.
Data exclusions	Data availability was limited prior to 1960 (especially across latitude), why only data records from 1960 onwards were analyzed. Data up to 2014 were considered to permit five-year blocking of the data and to investigate the same timespan across all four datasets.
Reproducibility	Latitudinal patterns of fish predation were analyzed independently for every ocean basin. Multiple statistical approaches were used to test robustness of results. We investigated and statistically accounted for a number of potential factors that could introduce bias to our results.
Randomization	We accounted for the repeated nature of the data by adding 'grid cell' as random effect into our GAMMs. We further accounted for spatial and temporal autocorrelation that is present in this type of data (see Methods for details and the exact GAMM syntax used to produce the main result).
Blinding	Blinding was not relevant to our study. Data were not collected with the specific purpose of measuring differences in predation across latitudes

Did the study involve field work? ☐ Yes ☒ No

Reporting for specific materials, systems and methods

We require information from authors about some types of materials, experimental systems and methods used in many studies. Here, indicate whether each material, system or method listed is relevant to your study. If you are not sure if a list item applies to your research, read the appropriate section before selecting a response.

Materials & experimental systems

n/a	Involved in the study
☒	☐ Antibodies
☒	☐ Eukaryotic cell lines
☒	☐ Palaeontology
☐	☒ Animals and other organisms
☒	☐ Human research participants
☒	☐ Clinical data

Methods

n/a	Involved in the study
☒	☐ ChIP-seq
☒	☐ Flow cytometry
☒	☐ MRI-based neuroimaging

Animals and other organisms

Policy information about studies involving animals; ARRIVE guidelines recommended for reporting animal research

Laboratory animals	No laboratory animals were used for this study
Wild animals	Catch records of multiple large predatory fish species in the open ocean were analyzed. Details on species are provided in Table S3. No scientific experiments on wild animals were performed for this study.
Field-collected samples	No scientific field collection was done for this study
Ethics oversight	NA (Data are publicly available and were not obtained specifically for this study)

Note that full information on the approval of the study protocol must also be provided in the manuscript.
