# Supplementary material for: Pelagic fish predation is stronger at temperate latitudes than near the equator
Source: Nat Commun. 2020 Mar 31;11:1527. doi: 10.1038/s41467-020-15335-4 (PMC7109113; doi:10.1038/s41467-020-15335-4)
Supplement: Supplementary file 4 — Description of Additional Supplementary Files [file 41467_2020_15335_MOESM4_ESM.pdf]

Description of Additional Supplementary Files

File Name: Supplementary Data 1
Description: Supplementary Data 1 provides the data underlying Figs 1-4, Supplementary Table 3 and Supplementary Figs 1,2,7-13,15,19-22, including different estimates of relative predation, hook saturation, total number of predators caught and hooks set. Further details are provided in the 'Read Me' as part of the Supplementary Data 1 workbook.

File Name: Supplementary Software 1
Description: Supplementary Software 1 provides the R-codes for initial processing and filtering of the raw longline data (c_DataPrep_allOceans.r), to add ocean depth (c_Ocean.depth.r) and distance to land (c_Distance.to.land.r) to the longline data, and to obtain and visualize the results shown in Figure 1 (c_Main.Analysis.r).
